# The Association between Regional Environmental Factors and Road Trauma Rates: A Geospatial Analysis of 10 Years of Road Traffic Crashes in British Columbia, Canada

**DOI:** 10.1371/journal.pone.0153742

**Published:** 2016-04-21

**Authors:** Jeffrey R. Brubacher, Herbert Chan, Shannon Erdelyi, Nadine Schuurman, Ofer Amram

**Affiliations:** 1 Department of Emergency Medicine, University of British Columbia, Vancouver, British Columbia, Canada; 2 Department of Geography, Simon Fraser University, Burnaby, British Columbia, Canada; Beihang University, CHINA

## Abstract

**Background:**

British Columbia, Canada is a geographically large jurisdiction with varied environmental and socio-cultural contexts. This cross-sectional study examined variation in motor vehicle crash rates across 100 police patrols to investigate the association of crashes with key explanatory factors.

**Methods:**

Eleven crash outcomes (total crashes, injury crashes, fatal crashes, speed related fatal crashes, total fatalities, single-vehicle night-time crashes, rear-end collisions, and collisions involving heavy vehicles, pedestrians, cyclists, or motorcyclists) were identified from police collision reports and insurance claims and mapped to police patrols. Six potential explanatory factors (intensity of traffic law enforcement, speed limits, climate, remoteness, socio-economic factors, and alcohol consumption) were also mapped to police patrols. We then studied the association between crashes and explanatory factors using negative binomial models with crash count per patrol as the response variable and explanatory factors as covariates.

**Results:**

Between 2003 and 2012 there were 1,434,239 insurance claim collisions, 386,326 police reported crashes, and 3,404 fatal crashes. Across police patrols, there was marked variation in per capita crash rate and in potential explanatory factors. Several factors were associated with crash rates. Percent roads with speed limits ≤ 60 km/hr was positively associated with total crashes, injury crashes, rear end collisions, and collisions involving pedestrians, cyclists, and heavy vehicles; and negatively associated with single vehicle night-time crashes, fatal crashes, fatal speeding crashes, and total fatalities. Higher winter temperature was associated with lower rates of overall collisions, single vehicle night-time collisions, collisions involving heavy vehicles, and total fatalities. Lower socio-economic status was associated with higher rates of injury collisions, pedestrian collisions, fatal speeding collisions, and fatal collisions. Regions with dedicated traffic officers had fewer fatal crashes and fewer fatal speed related crashes but more rear end crashes and more crashes involving cyclists or pedestrians. The number of traffic citations per 1000 drivers was positively associated with total crashes, fatal crashes, total fatalities, fatal speeding crashes, injury crashes, single vehicle night-time crashes, and heavy vehicle crashes. Possible explanations for these associations are discussed.

**Conclusions:**

There is wide variation in per capita rates of motor vehicle crashes across BC police patrols. Some variation is explained by factors such as climate, road type, remoteness, socioeconomic variables, and enforcement intensity. The ability of explanatory factors to predict crash rates would be improved if considered with local traffic volume by all travel modes.

## Introduction

Worldwide, over 3000 people per day are killed in road trauma, many more are injured and disabled, and the problem is increasing.[1] Road safety is closely linked to the cultural, political, and physical environments. Within high income countries, the road trauma burden is borne disproportionately by people living in poorer regions.[2, 3] The rates of pedestrian[4] and child pedestrian fatalities[5–7] are higher in poor neighbourhoods, possibly due to differences in risk taking, increased foot travel, and differences in the built environment (high speed roads, traffic calming measures, safe crosswalks, or other pedestrian safety features).[2, 3] In most developed countries, rural and remote regions have higher road fatality rates than urban areas. This discrepancy may be partially explained by features of the built and geographic environment: high speed roads with fewer crash mitigation features, harsher climate, difficulty enforcing traffic laws in remote regions, increased “discovery time” after a crash, and limited access to trauma care.[8–14] Cultural factors, such as public acceptance of risky driving, also influence the road trauma rate. Drivers are more likely to exceed the speed limit and less likely to wear seatbelts on rural than on urban roads.[8, 15]

British Columbia (BC), Canada covers almost a million square kilometers and includes large metropolitan centres as well as vast tracts of sparsely populated wilderness. Although the road fatality in BC is declining[16] it unfortunately remains above the Canadian average (5.8 per 100,000), and is over twice that in countries with the safest roads.[17–21] Traffic enforcement in BC is provided by 118 police patrols including 12 independent municipal jurisdictions and 106 Royal Canadian Mounted Police (RCMP) jurisdictions. Some jurisdictions have dedicated traffic units. In others, traffic enforcement is provided as part of the regular duties of all uniformed members. In addition British Columbia has 24 Integrated Road Safety Units (IRSU) which provide traffic enforcement to larger regions that overlap with the smaller jurisdictions.[22] Across the areas served by these patrols, there is marked variability in climate, socio-economic status, per capita alcohol consumption, access to trauma care, predominant road type, and other factors that could influence road safety outcomes.

### Objectives

In this cross-sectional study, we examine variation in type and severity of crashes across police patrols in BC and investigate potential explanatory factors that might explain this variation. We study the total number of crashes, injury crashes, single vehicle night-time crashes, rear end crashes, and crashes involving pedestrians, cyclists, motorcyclists, and heavy vehicles. We also study fatal crashes, number of fatalities, fatal crashes related to alcohol, and fatal crashes related to speeding. For potential explanatory factors we consider intensity of traffic law enforcement, speed limits, climate, remoteness and access to trauma care, socio-economic factors, and alcohol consumption.

### Methods

This study involved analysis of anonymized data. Informed consent was not obtained. the study was approved by the research ethics board of the University of British Columbia, Vancouver, British Columbia. We obtained crash outcomes from police reports (2003–2012; n = 386,326) and insurance claims (2000–2012; n = 1,434,239).

### Police reports

Police are obligated to attend all fatal crashes in BC. Police reports are reconciled with coroner’s data to ensure that all fatal crashes and all fatalities are captured. Police also attend most serious injury crashes but not all minor injury or property damage only crashes. Police reports include location and date of crash as well as factors that contributed to the crash (e.g. alcohol impairment, speeding, distraction). They also identify crashes involving vulnerable road users. We used police reports to identify fatal crashes due to alcohol, speeding, or distraction, as well as fatal crashes involving vulnerable road users (pedestrians, pedal cyclists, and motorcyclists) or heavy vehicles (e.g. transport truck, bus).

### Insurance Claims

The Insurance Corporation of British Columbia (ICBC) is the sole provider of basic automobile insurance in BC. All crashes that involve a BC registered vehicle and result in an insurance claim are reported to ICBC. Claims are based on driver’s reports and include date and location of crash, crash configuration, crashes involving vulnerable road users (pedestrians, pedal cyclists, and motorcyclists), and crashes involving a heavy vehicle. We excluded out-of-province incidents, vandalism, and incidents occurring in a parking lot. Because they are self reported, claims do not include contributory factors such as impaired driving. We used single vehicle night-time crashes (SVNCs) as an indicator of alcohol related crashes. Impaired drivers are over-represented in SVNCs,[23, 24] and SVNCs are often used as a surrogate for alcohol related crashes.[25–30] Table 1 (below) lists the crash events studied in this report.

**Table 1:** Crash events. This table summarizes annual average crash rates per 10,000 licensed drivers† for important crash events (2003–2012). Crash events not included in the analysis because of low rates are indicated in parentheses.

Event type	Provincial Average	Police Patrols						Comments
		Average^§^ (SD)	Min	Q1^¶^	Median	Q3^¶^	Max	
*Police reported crashes from Traffic Accident System (TAS) per 10*,*000 drivers per year*								
Fatal crashes	1.1	3.0 (3.7)	0.0	0.9	1.5	3.6	23.0	All crashes with a fatality
Fatalities^†^	27	87 (102)	0	29	56	106	650	All fatalities
(Fatal alcohol)	0.3	0.8 (1.1)	0.0	0.2	0.4	1.2	5.4	Fatal crashes with alcohol as contributory factor
(Fatal distraction)	0.3	0.7 (1.3)	0.0	0.1	0.3	0.8	8.2	Fatal crashes with distraction as contributory factor
Fatal speeding	0.4	1.2 (1.8)	0.0	0.2	0.6	1.2	12.5	Fatal crashes with speeding as contributory factor
*Claims per 10*,*000 drivers per year*								
All crashes	475	404 (212)	0	258	384	499	1140	Counts all insurance claims for a collision
Injury crashes	151	93 (55)	11	57	77	120	249	Only claims involving an injury
Single vehicle night-time crashes	36.7	63.9 (49.8)	6.0	28.5	45.4	87.6	221.0	Claims for a single vehicle crash between 9 PM and 6 AM–a surrogate for alcohol related crashes
Rear end crashes	168	59 (72)	0	14	30	68	334	Claims for a rear end collision–a surrogate for distraction related crashes
Pedestrian claims^‡^	3.9	1.9 (1.5)	0.0	0.9	1.6	2.7	8.0	Claims where a pedestrian was injured
Cyclist crashes^‡^	2.3	0.9 (1.1)	0.0	0.2	0.6	1.3	7.6	Claims where a pedal cyclist was injured in an MVC
Motorcyclist crashes	4.9	4.3 (5.5)	0.0	2.1	3.4	5.5	48.9	Claims for a motorcyclist crash
Heavy Vehicle crashes	29	39 (46)	0	10	23	49	248	Claims for a heavy crash

† Fatalities are reported as an annual average rate per 10,000 crashes.

‡ Pedestrian and cyclist crashes are reported as an annual average rate per 10,000 population.

§This is an average of the annual averages across police patrols. It gives equal weight to each patrol, while the provincial average is based on the total population of BC.

Q1, and Q3 denote the lower quartile, and upper quartile, respectively.

### Potential Explanatory Factors

For each police patrol, we considered the following factors that might affect the number, type, and severity of crashes: i) intensity of traffic law enforcement, ii) speed limits, iii) climate, iv) remoteness and access to trauma care, v) socio-economic factors, and vii) alcohol consumption.

To measure intensity of traffic law enforcement, we examined the rate of total traffic contraventions per 1,000 licensed drivers from 2003 to 2011. We also considered separate categories for contraventions related to alcohol and speeding, but those variables were deemed redundant due to high correlation with total contraventions. We examined the number of dedicated traffic officers in each police patrol (2006 to 2011), but decided not to use this variable due to data reliability issues. Instead we categorized police patrols according to whether or not they had a dedicated traffic unit. We measured highway infrastructure using the proportion of low (≤ 60 km/hr), medium (70 or 80 km/hr) and high (≥100 km\hr) speed roads in police patrols in 2011. Speed limits are estimated based on road type and population density using CanMap RouteLogistics^®^ v. 2011.3 (DMTI Spatial Inc, Markham, Ontario). We considered categorizing patrols according to proportions of road in urban/rural areas but this was highly correlated with speed limit, and both measures were not needed. Similarly, we considered several measures of climate that were highly correlated. Since we expect variation in winter weather to be highly related to the number and severity of crashes in BC, we restricted our analysis of climate to total winter precipitation (based on climate normal from 1981 to 2010) and average monthly winter temperature (based on climate normal from 1971 to 2000). For remoteness and access to trauma care, we examined the proportion of the population within four hours of a level 1 or 2 trauma centre for each police patrol. We considered using 1 or 2 hours from a trauma centre but these variables were highly correlated with low road speed and, compared to the 4-hour variable, were generally more correlated with other variables included in the analysis. The Vancouver Area Neighbourhood Deprivation Index (VANDIX) score was used as a measure of socio-economic factors. The VANDIX score is a linear combination of lone parent percentage, home owner percentage, average income, labour force participation rate, unemployment rate, percentage without a high school education, and percentage with a university education. Higher scores indicate poorer socioeconomic indicators.[31] For alcohol consumption we examined alcohol sales and liquor licenses (2013) per population aged 15 and up in each police patrol using population estimates from the 2010 census.

### Mapping

We obtained shapefiles for each police patrol. Police reported crashes and automobile insurance claims were mapped to police patrol using a combination of city of event, postal code, or GIS coordinates as available. We used CanMap RouteLogistics^®^ v. 2011.3 (DMTI Spatial Inc, Markham, Ontario) and methods appropriate to available data to map potential explanatory factors and crash events to police patrol. After mapping crashes to police patrols, we generated annual per capita counts for each crash event and each police patrol.

### Analysis

We selected eleven outcomes for analysis. From ICBC insurance claims we looked at eight outcomes: total crashes, injury crashes, SVNCs, rear-end collisions, collisions involving heavy vehicles, collisions involving pedestrians, collisions involving cyclists, and collisions involving motorcyclists. From police reports we studied total fatal crashes, speed related fatal crashes, and total fatalities. There were too few alcohol related fatal crashes per patrol (only 7, on average, during the 10-year study period) for meaningful analysis. We did not study police reports of non-fatal crashes because reporting of these crashes is discretionary and reporting practice likely varies across police jurisdictions.

For all crash types, except those involving pedestrians or cyclists, we computed annual rates per 1000 licensed drivers for each police patrol. For crashes involving pedestrians or cyclists, we calculated rates per entire population. Seventeen police patrols with fewer than 1000 drivers were excluded from our analysis. One additional patrol was excluded because of an apparent error in the raw data resulting in an over-reporting of claims. Therefore our analysis is based on 100 police patrols. Rates, for each patrol, represent the number of crash events for the entire study period (2003 to 2012). We also computed the fatality rate per 10,000 crashes.

Factors suspected to be associated with crashes were selected a priori based on literature review. We chose 6 categories of factors (intensity of traffic law enforcement, roadway speed limit, climate, access to trauma care, socio-economic factors, and alcohol consumption) and chose one or two variables of interest from each category to act as covariates in our models. To avoid over fitting, we eliminated highly correlated factors. The variance inflation factor, a measure of multicollinearity, was less than four for all selected predictors. Table 2 (below) lists the factors included in our models.

**Table 2:** Explanatory Factors. This table lists the potential explanatory factors included in the models and summarizes their variability across police jurisdictions.

Factor	Police Patrols						Comments
	Average (SD)	Min	Q1^§^	Median	Q3^§^	Max	
*Enforcement*							
Citations per driver	274 (234)	26	157	207	299	1645	Contravention rate per 1,000 drivers, 2003 to 2011
Dedicated traffic officers*	0.5						Indicator for whether there were actual dedicated traffic officers at any time from 2006 to 2011
*Climate*							
Winter temperature (°C)	1.0 (4.7)	-11.2	-2.6	-0.5	5.8	8.2	Mean of monthly maximum temperature in winter months (Dec to Feb) based on 1981–2010 Climate Normals published by Environment Canada
Winter precipitation (mm)	407 (288)	74	165	331	590	1280	Total precipitation during winter months (Dec to Feb) based on 1971–2000 Climate Normals published by Environment Canada
*Socio-economic factors*							
Lone parent (%)	27.8 (6.2)	15.2	23.4	27.1	31.9	48.8	Taken directly from or calculated from DA 2006 Census profile tables
Home owner (%)	73.8 (10.2)	41.9	71.4	76.5	80.4	90.1	Taken directly from or calculated from DA 2006 Census profile tables
Average income ($)	32126 (7086)	16832	28793	30972	35716	74134	Taken directly from or calculated from DA 2006 Census profile tables
Population in workforce (%)	65.1 (7.4)	48.9	59.5	65.6	70.0	84.2	Taken directly from or calculated from DA 2006 Census profile tables
Unemployment rate (%)	8.6 (4.7)	1.9	5.4	7.2	10.7	28.8	Taken directly from or calculated from DA 2006 Census profile tables
No high school education (%)	25.9 (9.1)	8.1	19.5	25.5	30.5	57.4	Taken directly from or calculated from DA 2006 Census profile tables
University education (%)	17.5 (9.6)	6.5	11.5	14.4	19.2	65.9	Taken directly from or calculated from DA 2006 Census profile tables
VANDIX score^†^	0.00 (0.74)	-1.79	-0.38	-0.04	0.27	2.36	Vancouver Area Neighbourhood Deprivation Index
*Alcohol consumption*							
Alcohol sales per capita	11.2 (3.1)	4.0	9.7	10.8	12.7	23.2	Alcohol sales in liters of absolute alcohol per 15+ capita, 2010
Alcohol serving establishments per capita	4.4 (3.4)	0.0	2.3	3.4	5.2	19.5	Number of liquor licenses per 1,000 population 15+, March 2013
*Remoteness and access to trauma care*							
4 hrs to trauma centre (%)	61.4 (47.8)	0.0	0.0	99.8	100.0	100.0	Percent of the population within four hours of a level 1 or 2 trauma centre
*Highway infrastructure*							
High speed roads (%)	6.4 (8.1)	0.0	2.1	4.6	7.7	50.1	Percent of total kilometers of road with 100 km/h speed limit in 2011
Low speed roads (%)	18.4 (32.8)	0.0	0.0	0.7	15.6	100.0	Percent of total kilometers of road with 50 km/h or 60 km/h speed limit in 2011

* The average represents the proportion of police patrols with dedicated traffic officers.

† The VANDIX score is a linear combination of all other socio-economic factors.

§ Q1 and Q3 denote the lower quartile and upper quartile, respectively.

To accommodate lack of normality and overdispersion in the data, we modelled per capita counts for each outcome using negative binomial regression. We explored Poisson models, but found that negative binomial models fit the data better based on chi-square tests of the residual deviance and degrees of freedom. In our negative binomial models, each response variable is the crash count in each police patrol. We also included an offset term for the population at risk in each police patrol during the study period. All selected explanatory factors were included as covariates in each crash outcome model. Since we used the same covariates in each model, we adjusted our p-values for multiple comparisons using the Bonferroni correction. For example, we tested the null hypothesis that winter precipitation is not associated with crash events for eleven different event types, therefore we multiplied the resulting p-values by eleven. Although conservative, the Bonferroni correction is simple to apply and easily understood. In light of the exploratory nature of this analysis, a more complex adjustment was not necessary.

## Results

### Crash types

**Fig 1:**
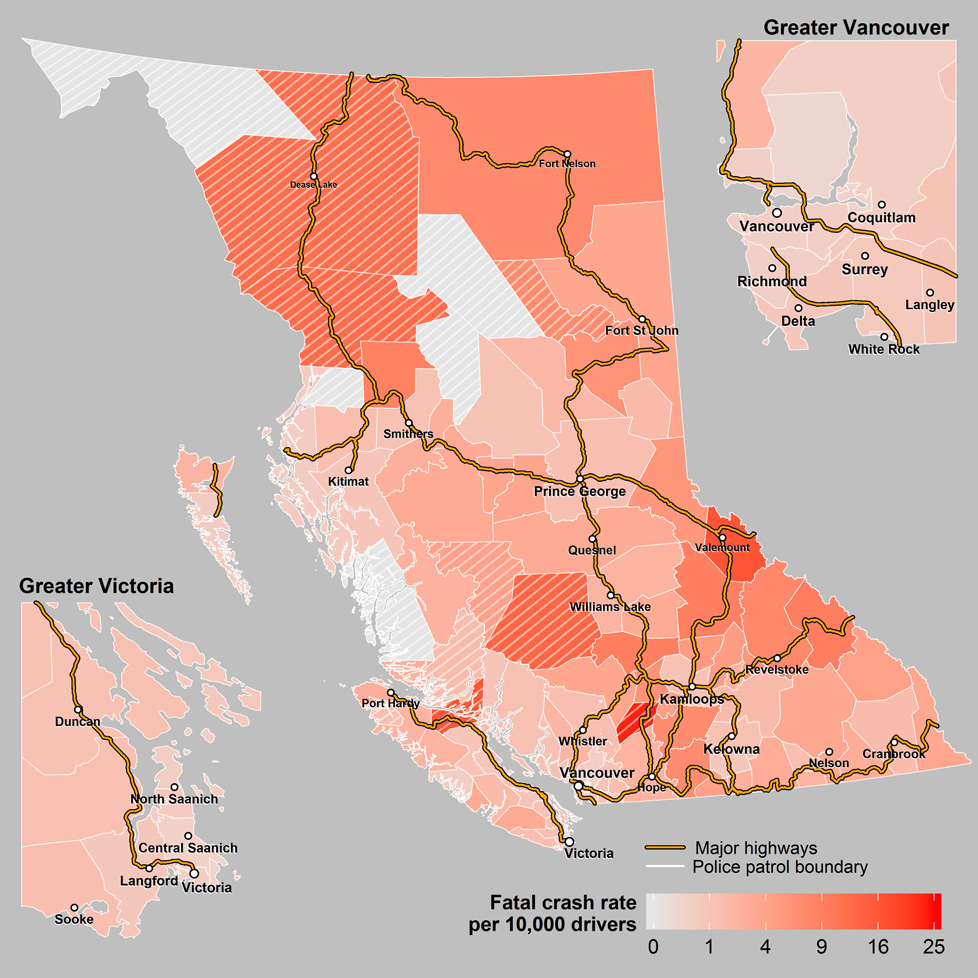
Fatal Crashes. Annual rates of fatal crashes across police patrols in BC. The cross hatched areas represent sparsely populated patrols where the rates may be unstable.

**Fig 2:**
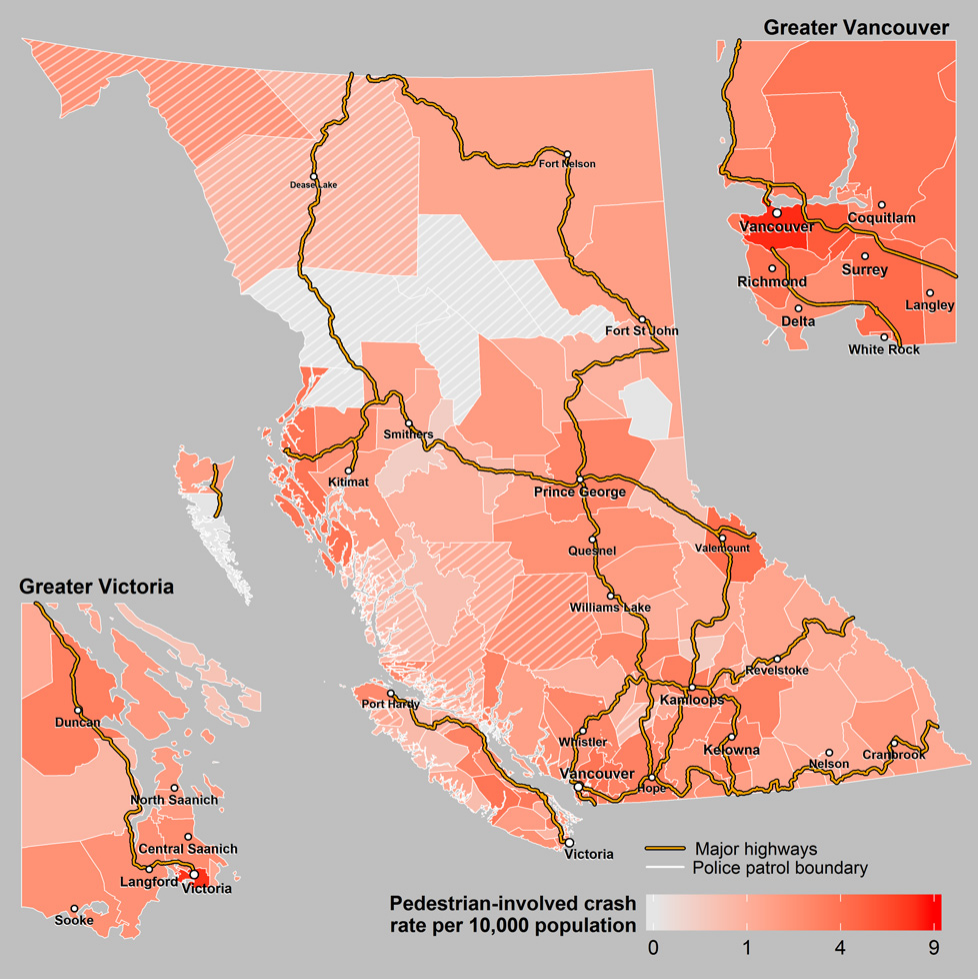
Pedestrian Crashes. Annual rates of crashes involving a pedestrian across police patrols in BC. The cross hatched areas represent sparsely populated patrols where the rates may be unstable.

**Fig 3:**
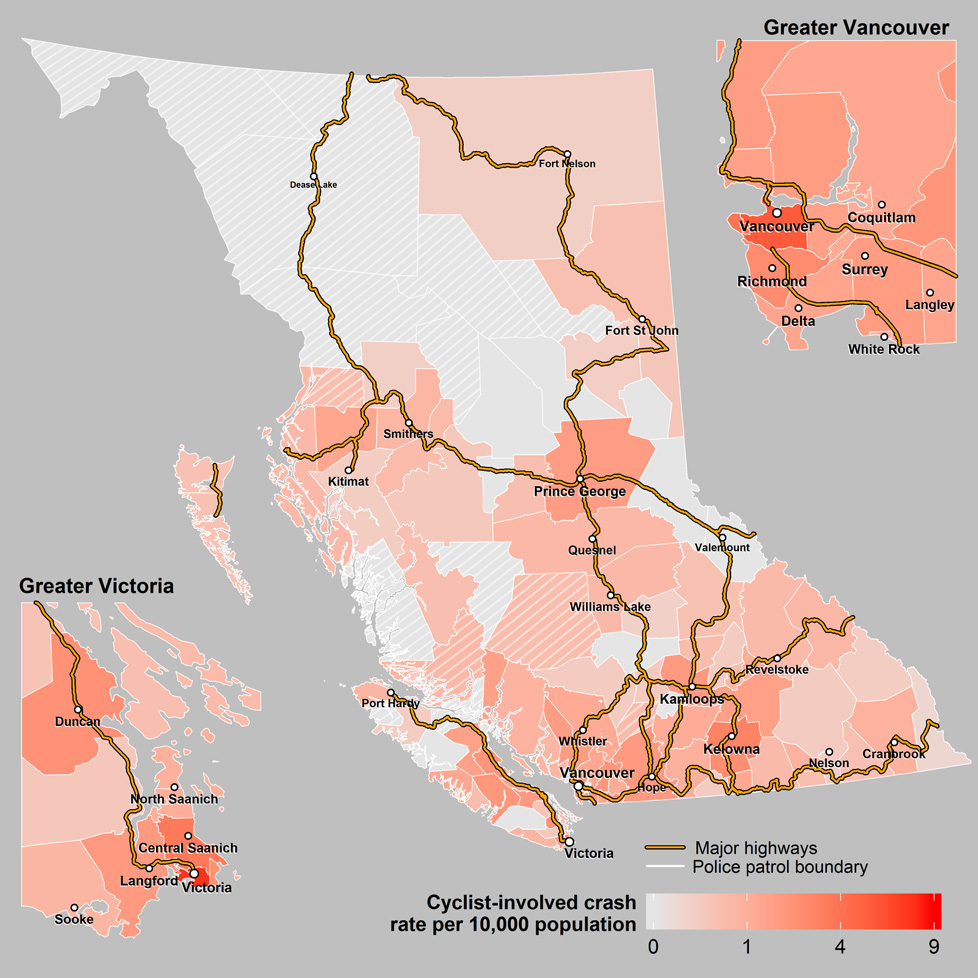
Cyclist Crashes. Annual rates of crashes involving a cyclist across police patrols in BC. The cross hatched areas represent sparsely populated patrols where the rates may be unstable.

**Fig 4:**
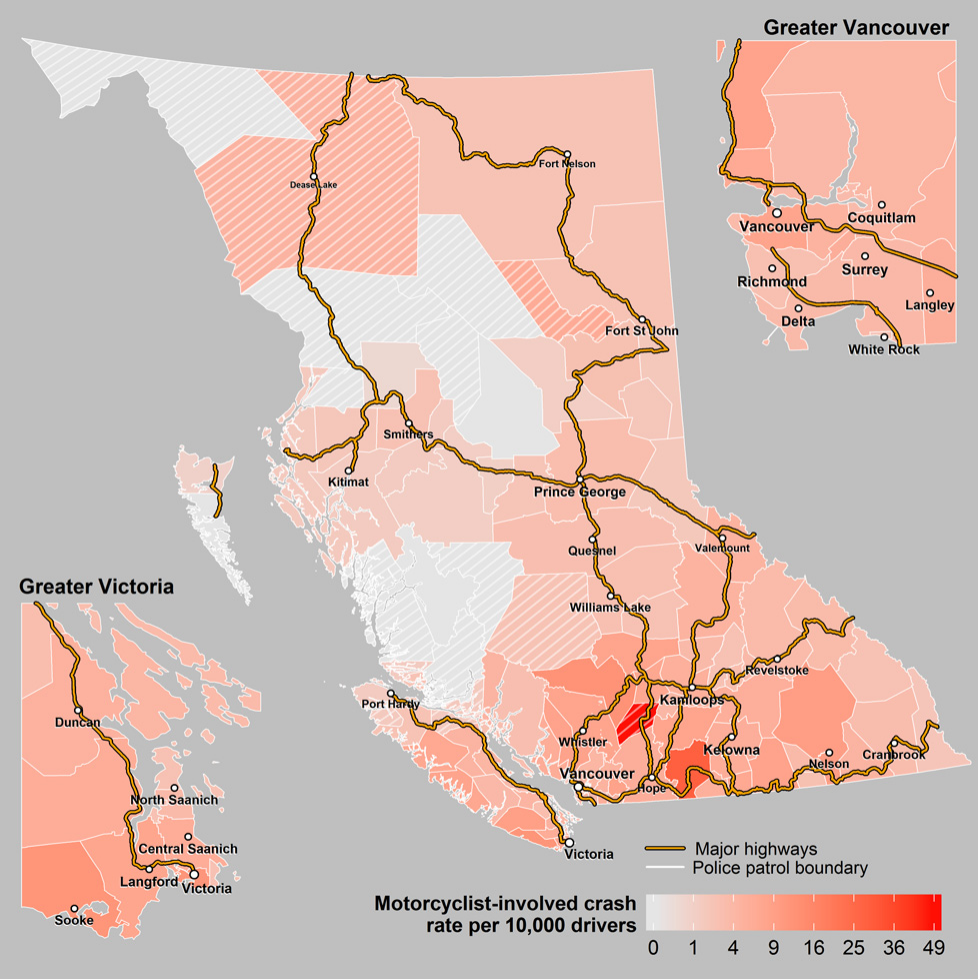
Motorcyclist crashes. Annual rates of crashes involving a motorcyclist across police patrols in BC. The cross hatched areas represent sparsely populated patrols where the rates may be unstable.

Over the 10 year study period, there was an annual average of 143,424 insurance claim collisions and 38,633 police reported crashes which included 1,710 incidents involving pedestrians (63 were fatal), 1,010 incidents involving pedal cyclists (9 fatal), 1,494 incidents involving motorcyclists (39 fatal) and 8,792 incidents involving heavy vehicles (63 fatal). There was an annual average of 340 fatal crashes resulting in 381 fatalities. For all crash events, there were large differences in the per capita rate across the 118 police patrols (Table 1, Figs 1–4).

### Explanatory Factors

**Fig 5:**
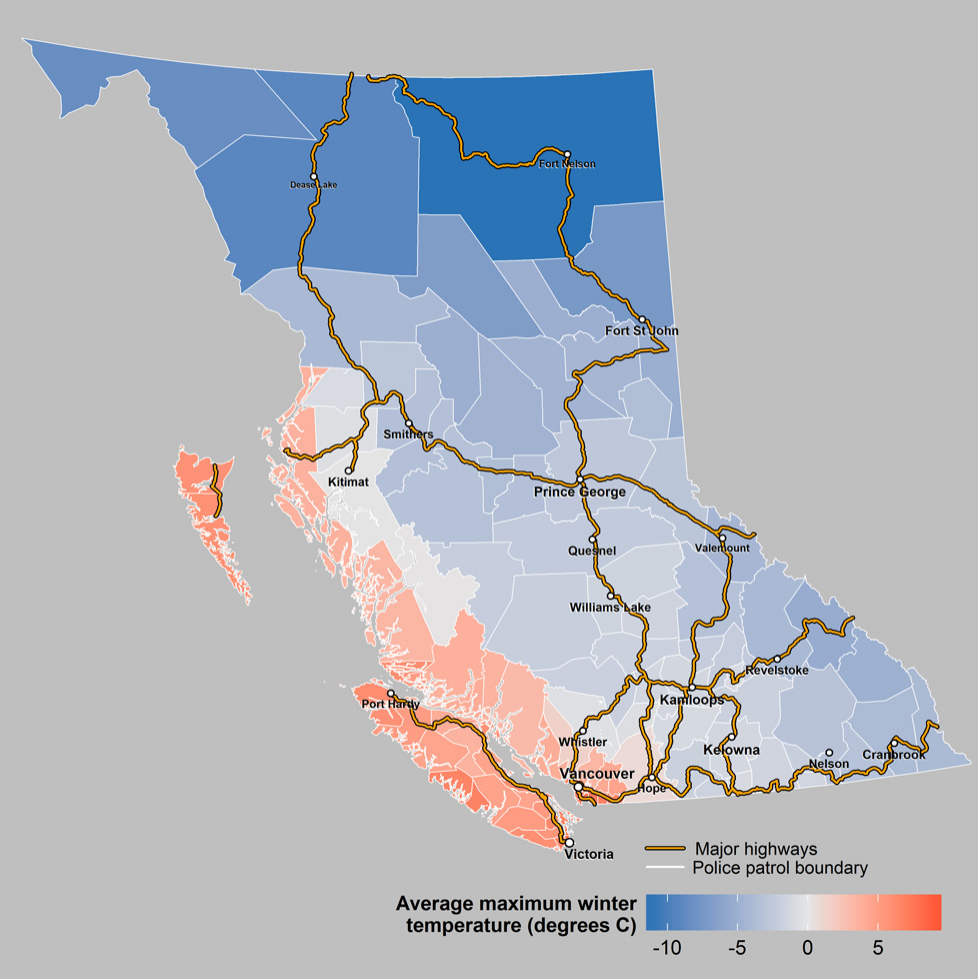
Winter Temperature. This figure shows variation in winter temperatures across BC’s police patrols.

**Fig 6:**
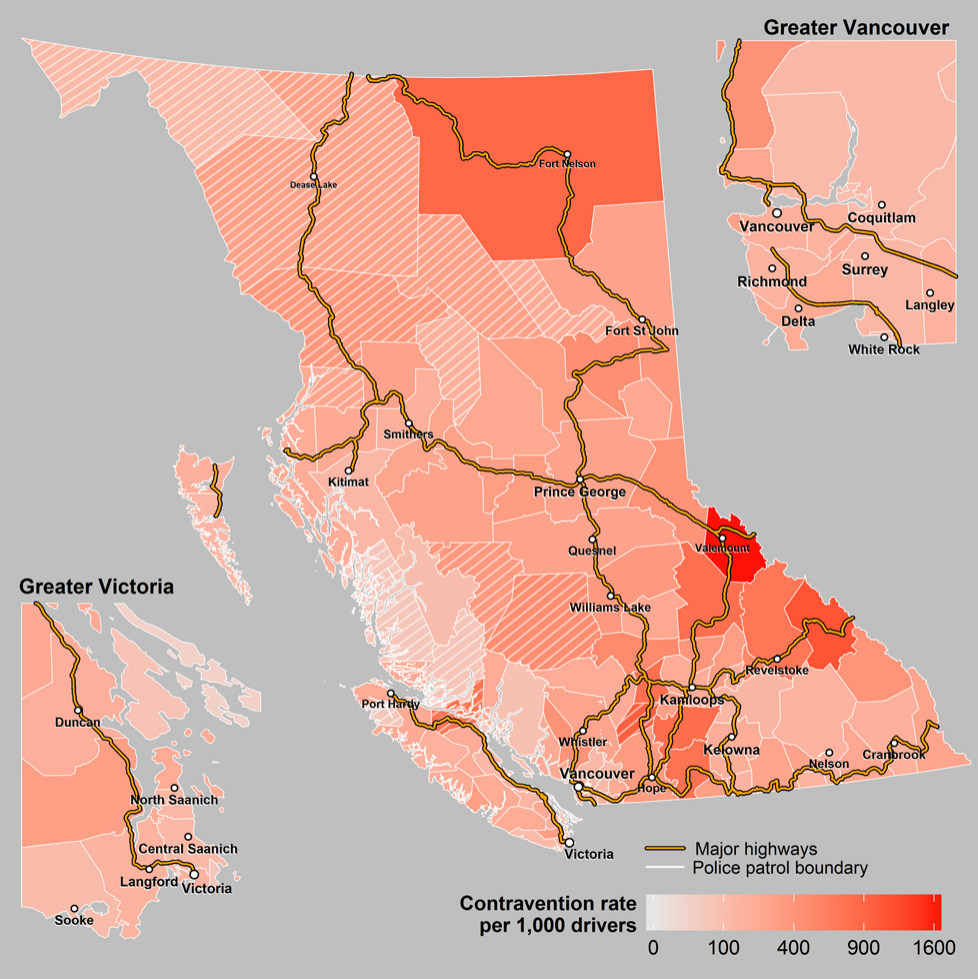
Driving Contraventions. This figure shows the number of driving contraventions issued per 1,000 licensed drivers according to police patrol. The cross hatched areas represent sparsely populated patrols where the rates may be unstable.

**Fig 7:**
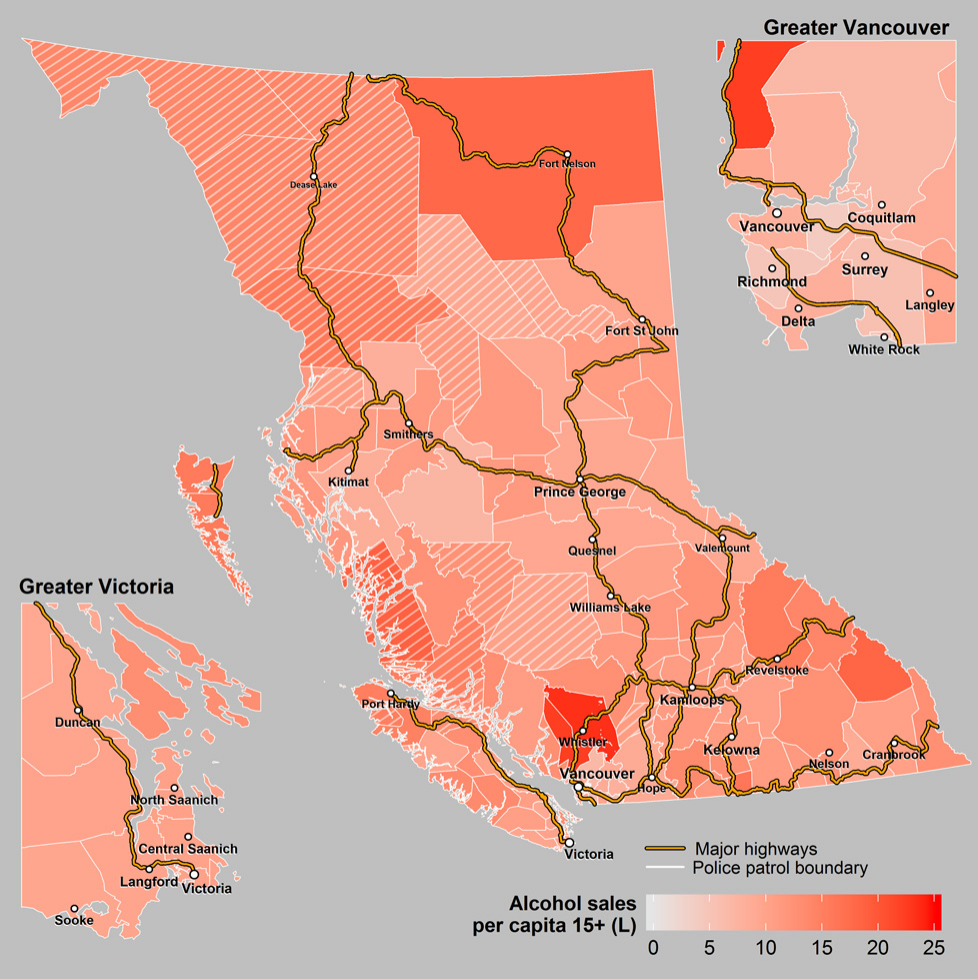
Alcohol Sales. This figure shows alcohol sales in litres per population ≥15 years. The cross hatched areas represent sparsely populated patrols where the rates may be unstable.

There was marked variation in potential explanatory factors across the province. The annual number of traffic citations per 1,000 licenced drivers in each police patrol ranged from 26 to 1,645. The highest number of per capita citations occurred in sparsely populated patrols (that were excluded from the regression analysis because of small population) and likely reflect citations given to drivers from outside the jurisdiction. Maximum average winter temperature ranged from -11.2 to 8.2 degrees Celsius, and winter precipitation ranged from 74 to 1280 mm. The composite VANDIX scores ranged from -1.79 to 2.36 in which higher scores indicate poorer socioeconomic status. Percent of population within four hours of a trauma centre, and percent of roadways comprised of highway and city speed limits all ranged from 0 to 100%. There was also a wide variation in per capita alcohol sales and licensed alcohol outlets across police patrol jurisdictions ranging from 4 to 23.2 liters per capita (age ≥ 15) and 0 to 19.5 premises per 1000 population age ≥ 15 respectively (Table 2, Figs 5–7).

### Correlation between crash types and explanatory factors

**Fig 8:**
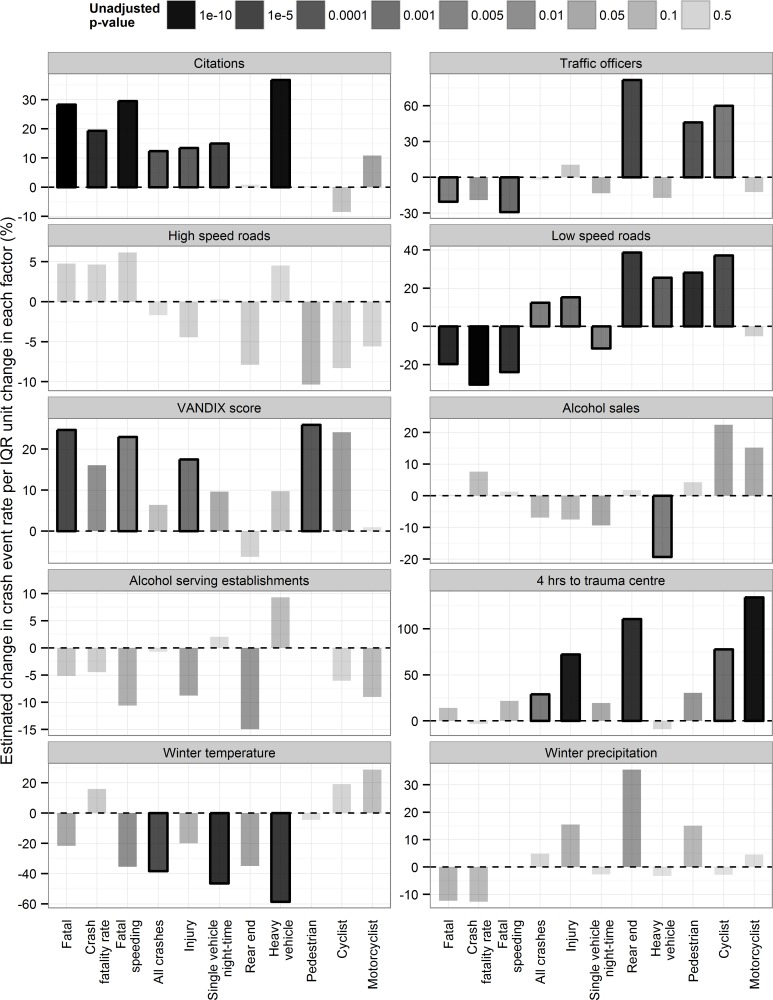
Relationship between crash type and explanatory factors. This figure uses vertical bars to show the association between predictive factors and crash outcomes. The shade correlates with unadjusted p values (deeper shade = smaller p values), solid outlines indicate relationships that remain significant after correction for multiple comparisons (Bonferroni). The height of the bars indicate the percentage change in crash events per unit change in predictive factor, where one unit is the interquartile range for that factor.

The results from negative binomial regression analyses illustrate the relationships between selected explanatory factors and each crash type (Table 3, Fig 8). Alcohol sales per capita were significantly associated with lower rates of collisions involving heavy vehicles. Licensed alcohol outlet density was not significantly associated with any collision type. Percent of population within four hours of a trauma centre was significantly associated with overall collisions, rear end collisions, collisions involving cyclists, and collisions involving motorcyclists. As expected, percent of roadways comprised of city speed limits (≤ 60 km/hr) was positively associated with rear end collisions, and collisions involving pedestrians, cyclists, and heavy vehicles but was negatively associated with SVNCs, fatal crashes, and fatalities. Conversely, percentage highway speed roadways (≥ 100 km/hr) had no independent statistically significant relationship with any crash type. Higher average winter temperature was significantly associated with lower rates of overall collisions, SVNCs, and heavy vehicles collisions. For every degree Celsius increase, the rates of overall crashes, SVNCs, heavy vehicles collisions, and speed related fatal collisions decreased by 5.3%, 6.7%, 10.2%, and 6.1%, respectively. Winter precipitation had no significant relationship with any collision type. Traffic contravention rates were positively associated with overall collisions, injury crashes, SVNCs, fatal crashes, speed related fatal crashes, total fatalities, and collisions involving heavy vehicles but not with rear end collisions nor with collisions involving vulnerable road users. Having designated traffic enforcement officers was positively associated with rear end collisions, and collisions involving pedestrians and cyclists, and negatively associated with fatal crashes and speed related fatal crashes. Lower socio-economic status (higher VANDIX scores) was positively associated with collisions involving pedestrians, injury collisions, fatal crashes, and speed related fatal crashes.

**Table 3:** Association between predictive factors and crashes: This Table reports unadjusted p-values and summarizes the percent change in rate in crash event per unit change in each factor as estimated from the models. The units for each predictive factor are normalized so that one unit equals the interquartile range for that factor.

		Percent change in event rate per IQR unit change in each factor (unadjusted p-value)					
*Factor*	*Unit Change (IQR)*^*‡*^	*Fatal*	*Crash fatality rate*	*Fatal speeding*	*All crashes*	*Injury*	*Single vehicle night-time*
Intercept^†^		1.9 (<0.001)*	57.0 (<0.001)*	0.7 (<0.001)*	388.2 (<0.001)*	98.4 (<0.001)*	57.4 (<0.001)*
Citations	137 per 1,000 drivers	28.3 (<0.001)*	19.3 (<0.001)*	29.5 (<0.001)*	12.3 (<0.001)*	13.5 (<0.001)*	15.0 (<0.001)*
Traffic officers	1 (indicator)	-20.5 (0.004)*	-19.2 (0.018)	-29.2 (0.001)*	-1.9 (0.806)	10.5 (0.258)	-13.5 (0.086)
High speed roads	4.7%	4.8 (0.364)	4.6 (0.435)	6.2 (0.377)	-1.7 (0.720)	-4.5 (0.390)	0.3 (0.956)
Low speed roads	23.1%	-19.7 (<0.001)*	-30.4 (<0.001)*	-23.8 (<0.001)*	12.4 (0.003)*	15.3 (0.001)*	-11.5 (0.004)*
VANDIX score	0.6	24.7 (<0.001)*	16.1 (0.011)	22.9 (0.004)*	6.4 (0.167)	17.5 (0.001)*	9.6 (0.056)
Alcohol sales	3.1L per pop 15+	0.3 (0.947)	7.6 (0.155)	1.3 (0.835)	-6.9 (0.117)	-7.5 (0.123)	-9.4 (0.044)
Alcohol serving establishments	2.1 per 1,000 pop 15+	-5.2 (0.236)	-4.5 (0.346)	-10.6 (0.067)	-0.7 (0.850)	-8.8 (0.033)	2.0 (0.627)
4 hrs to trauma centre	100%	14.0 (0.151)	-3.4 (0.733)	21.6 (0.110)	28.8 (0.002)*	72.2 (<0.001)*	19.3 (0.048)
Winter temperature	8.3°C	-21.7 (0.051)	15.9 (0.298)	-35.4 (0.010)	-38.3 (<0.001)*	-19.9 (0.086)	-46.5 (<0.001)*
Winter precipitation	425mm	-12.4 (0.099)	-12.7 (0.131)	-0.0 (1.000)	4.9 (0.511)	15.5 (0.074)	-2.7 (0.723)
*Factor*	*Unit Change (IQR)*^*‡*^	*Rear end*	*Heavy vehicle*	*Pedestrian*	*Cyclist*	*Motorcyclist*	* *
Intercept^†^		63.9 (<0.001)*	19.3 (<0.001)*	2.1 (<0.001)*	1.0 (0.774)	4.8 (<0.001)*	
Citations	137 per 1,000 drivers	0.8 (0.876)	36.7 (<0.001)*	0.3 (0.939)	-8.5 (0.204)	10.8 (0.035)	
Traffic officers	1 (indicator)	81.6 (<0.001)*	-17.4 (0.126)	46.0 (<0.001)*	60.0 (0.002)*	-12.5 (0.279)	
High speed roads	4.7%	-7.9 (0.331)	4.5 (0.557)	-10.3 (0.083)	-8.3 (0.390)	-5.6 (0.467)	
Low speed roads	23.1%	38.7 (<0.001)*	25.5 (<0.001)*	28.1 (<0.001)*	37.2 (<0.001)*	-5.2 (0.391)	
VANDIX score	0.6	-6.3 (0.414)	9.7 (0.194)	25.9 (<0.001)*	24.1 (0.025)	0.9 (0.903)	
Alcohol sales	3.1L per pop 15+	1.8 (0.827)	-19.3 (0.003)*	4.2 (0.471)	22.4 (0.031)	15.2 (0.051)	
Alcohol serving establishments	2.1 per 1,000 pop 15+	-15.0 (0.018)	9.3 (0.147)	-0.0 (0.999)	-6.1 (0.481)	-9.0 (0.132)	
4 hrs to trauma centre	100%	110.5 (<0.001)*	-9.1 (0.471)	30.4 (0.016)	77.8 (0.001)*	134.2 (<0.001)*	
Winter temperature	8.3°C	-35.0 (0.036)	-58.6 (<0.001)*	-4.4 (0.755)	19.1 (0.451)	28.7 (0.168)	
Winter precipitation	425mm	35.5 (0.017)	-3.3 (0.773)	15.1 (0.109)	-2.9 (0.835)	4.6 (0.692)	

* Statistically significant at a bonferroni adjusted level of 0.0045.

† The intercept represents the average event rate at the median level of all factors.

‡ Estimates for continuous factors are expressed per interquartile range (IQR) unit change in each factor. This can be loosely interpreted as the percent change in the event rate for regions with *typical low values* compared to regions with *typical high values* of each factor.

## Discussion

British Columbia is a large, geographically diverse jurisdiction with marked geographic variation in environmental and socio-cultural factors. The coastal regions have the warmest winters and most winter precipitation (Fig 5). The highest socio-economic scores are in the large urban centres with lower scores in the northern and interior regions of the province. The urban centres also have the highest percentage of low speed roads and generally also have dedicated traffic police. Per-capita traffic contravention rates are highest in the interior of the province, likely reflecting tickets issued to drivers from outside the region who travel along the major highways that cross these jurisdictions. The number of alcohol serving establishments per capita are highest in cities whereas per capita alcohol sales are generally higher outside of greater Vancouver. In some jurisdictions, especially tourist areas, per capita alcohol sales are likely inflated due to sales to persons from outside the region. There is also a wide range in the number and type of crashes per capita. The lowest overall crash rates are in BC’s coastal regions. BC’s large metropolitan centres, especially greater Vancouver and greater Victoria, have the lowest rates of fatal crashes and SVNCs but the highest rates of collisions involving pedestrians or cyclists, and the highest rates of rear end collisions.

### Alcohol sales and licenses

We hypothesized that jurisdictions with higher per capita alcohol sales and more alcohol licenses would have higher rates of crashes, especially SVNCs. Contrary to this assumption, we found that jurisdictions with higher alcohol sales had significantly lower rates of heavy vehicle crashes and, although not significant after Bonferroni correction, they also had fewer total crashes, fewer SVNCs, and more collisions involving a cyclist. In addition, the number of alcohol licenses per capita was not associated with any crash type. The negative relationship between alcohol sales and some crash types seems inconsistent with the well-known effect of drunk driving on crash risk. However, aggregated alcohol sales may be a poor indicator of the prevalence of drinking and driving in a community since individual factors associated with higher alcohol consumption, such as drinking culture, income, and education, are not necessarily associated with higher rates of drinking and driving in the region where alcohol is sold. The negative association between alcohol sales and risk of certain types of crashes may also be explained by the fact that several police patrols with the highest per capita alcohol sales are in tourist communities. Tourists are not counted in the census but likely account for a large portion of the alcohol sales in these communities, artificially increasing the apparent per capita alcohol sales. At the same time, tourist destinations may have better public transportation, more availability of taxis, and an infrastructure that supports walking or cycling–factors that could decrease the rate of certain collision types. The combination of artificially increased per capita alcohol sales combined with lower collision rates might explain the negative association between per capita alcohol sales and some collision types seen in this study. Similarly, the positive relationship between alcohol sales and collisions involving bicycles may be partly due to the fact that many tourist communities in British Columbia are frequented by recreational cyclists. On the other hand, very few transport trucks or heavy vehicles go through these communities, possibly explaining the negative relationship between alcohol sales and heavy vehicle crashes.

Other researchers have also investigated the association of alcohol outlet density and crash risk and found increased crash risk on roads[32, 33] or in regions[34] with higher density of alcohol serving establishments. Treno studied alcohol outlet densities over time and found that increases in outlet density were associated with higher rates of injury crashes and of police attended alcohol related crashes.[35] Other researchers found no association between alcohol outlet density and risk of crashes.[36, 37] Ponicki studied the association between alcohol outlet density, other retail outlet density, and motor vehicle crashes in California and found that higher general retail outlet density was associated with higher rates of injury crashes but the results were mixed regarding whether alcohol outlets had additional effects on injury crashes beyond their role as general retail outlets. They did find, however, that crashes occurring in areas with higher density of bars were more likely to be alcohol related, although the effect sizes were small.[26] The mixed results for the association between alcohol outlet density and crash risk are sometimes explained by the following theoretical consideration: higher density of alcohol outlets increase alcohol availability and thereby increase the number of impaired driving trips. At the same time, however, increasing alcohol outlet density reduces travel distances (to and from alcohol serving establishments) and thereby reduces the risk of collision per trip.

### Enforcement

Implementation and enforcement of evidence based traffic laws is one of the most effective ways of deterring dangerous driving and reducing road injuries.[38, 39] We hypothesized that jurisdictions with greater enforcement would have fewer SVNCs and fewer speed related crashes. In support of this hypothesis we found that designated traffic enforcement officers were negatively associated with fatal crashes and with fatal speed related crashes. The other associations between enforcement indicators and crashes are harder to explain. We found that traffic contravention rates were positively associated with overall collisions, injury crashes, SVNCs, fatal crashes, fatal crashes related to speeding, total fatalities, and collisions involving heavy vehicles. Similarly, designated traffic enforcement officers were positively associated with rear end collisions, and collisions involving pedestrians and cyclists. These associations could result if jurisdictions with heavier traffic or more crashes issue more traffic contraventions or are more likely to have dedicated traffic police–in other words, more crashes may “cause” more enforcement. Studying the effect of changes in enforcement over time on crash rates might overcome this problem.

### Remoteness and access to trauma care

We used percentage of the population with 4 hours of a trauma centre as both an indicator of access to trauma care, and an (inverse) indicator of remoteness. We hypothesized that regions with worse access to trauma care would have more fatal crashes and higher crash fatality rates. In contrast, we found a trend towards fewer fatal crashes per capita in remote regions.

Less remote regions also had significantly more total collisions, injury collisions, rear end collisions, collisions involving cyclists, and collisions involving motorcyclists. The higher numbers of rear end collisions in less remote jurisdictions is likely explained by higher traffic volumes. Similarly, cycling and motorcycling is likely more common in less remote regions, explaining the higher rates of collisions involving these road users.

### Road speed

We found that jurisdictions with a high prevalence of low speed roads (speed limit ≤ 60 km/hr) have lower rates of single vehicle night-time crashes, fatal crashes, fatal speed related crashes, and total fatalities. Jurisdictions with a high percentage of low speed roads are typically urban centres. Lower speed limits and lower traffic speed due to urban congestion likely reduce the incidence of fatal crashes and particularly fatal speed related crashes. Our finding of lower rates of SVNCs, a surrogate for alcohol related crashes, is consistent with other research demonstrating lower rates of alcohol related crashes in urban as compared to rural areas.[40, 41] Of course, not all SVNCs are alcohol related and the higher rate of SVNCs in rural regions may partially reflect worse road conditions or more driver fatigue in those regions. We also found that jurisdictions with more low speed roads had higher rates of total crashes, injury crashes, rear end crashes, and of crashes involving pedestrians, cyclists, or heavy vehicles–as would be expected in urban areas. We postulated that jurisdictions with more high speed roads would have higher crash fatality rates. However, the proportion of high speed roads (speed limit ≥ 100 km/hr) in each jurisdiction was not associated with any crash type. This may be explained by the fact that, although these roads are generally more dangerous than city streets, they may be safer than roads with speeds posted at 80 or 90 km/hr because of better design and maintenance.

### Socio-economic status

Consistent with our hypotheses, we found that poorer socioeconomic indicators were associated with increased rates of pedestrian involved collisions, injury collisions, fatal speeding collisions, and fatal collisions. Other investigators have found that, in developed countries, the road trauma burden is borne disproportionately by the poor,[2, 3] and that rates of pedestrian[4] and child pedestrian fatalities[5–7] are higher in poor neighbourhoods. In BC, children from low socio-economic families are over-represented in child pedestrian fatalities.[42]

### Climate

We found that regions with warmer winter temperatures had fewer total crashes, fewer SVNCs, and fewer heavy vehicle crashes. There was no association between winter precipitation and any crash outcome. Weather conditions may affect crash risk differently with a distinct geographical pattern across BC. Snowfall is more common in the mountainous North and Interior regions, while rain is typical in the southern region of lower mainland in winter months. Higher precipitation of any sort is thought to increase crash rates. However, some studies suggested that snowfall can be associated with a significant decline in road crashes, likely due to reduced traffic volume or lower travel speeds, whereas rainfall is liable to increase crashes.[43]

### Limitations

This study has several limitations. First, we are studying *association* and not cause and effect. This is particularly important when interpreting the relationship between enforcement intensity and crashes since jurisdictions may respond to high crash rates by increasing traffic law enforcement. The effect of traffic law enforcement on crash might be better studied by looking at the effect on crashes of increased enforcement in the same jurisdiction. In addition, since exposure and response variables in this study are measured only in aggregate, rather than for individuals, our study is also subject to the ecological fallacy which consists in thinking that relationships observed for groups necessarily hold for individuals.[44] Similarly, BC’s police patrols cover fairly large geographic areas, and explanatory factors identified at the patrol level are not necessarily the same as those that would be observed at a finer spatial scale. For example, although we found no relationship between the number of alcohol serving establishments in a police jurisdictions and any crash type, it is still possible that alcohol related crashes tend to occur close to alcohol serving establishments.

The predictive value of explanatory factors would likely be improved if considered in conjunction with local traffic volume by mode of transport (i.e. road exposure). As discussed above, the traffic volume in some patrols might be far greater than suggested by the number of drivers living in the patrol. For example, sparsely populated regions traversed by major thoroughfares may have falsely elevated per capita crash rates as a result of crashes involving people from outside the region. Similarly, jurisdictions with large numbers of travellers from outside the region may have inflated per capita rates of alcohol sales or traffic contraventions. The predictive value of other factors (e.g. alcohol outlets) might be improved if the analysis was done on a finer geographic scale to better reflect nearby events. Studying crashes on a finer geographic scale would be also required to investigate the road safety effect of local features of the built environment such as intersection design, roadside commerce, billboards,[45] or roadside memorials.[46, 47] In addition, as mentioned above, studying the effect of changes in enforcement over time might provide a better understanding of the effect of enforcement on crash rates. Finally, in order to simplify the modelling process and preserve interpretability, we assumed a linear relationship between all explanatory variables and the log of the expected response variables. The interaction terms of some of the explanatory factors needed to be considered, particularly interactions between temperature and precipitation as well as between alcohol consumption and outlet density.

## Conclusions

There is wide variation in per capita rates of motor vehicle crashes across police jurisdictions in BC. Some of this variation is explained by factors such as climate, road type, remoteness, socioeconomic variables, and enforcement intensity. Jurisdictions with a high prevalence of low speed roads (speed limit ≤ 60 km/hr) have lower rates of single vehicle nighttime crashes, fatal crashes, fatal speed related crashes, and total fatalities, but higher rates of rear end crashes and crashes involving pedestrians or cyclists. Poorer socioeconomic indicators were associated with increased rates of pedestrian involved collisions and of fatal collisions. Regions with warmer winter temperatures had fewer crashes overall, fewer single vehicle nighttime crashes, fewer fatal speeding crashes, and fewer heavy vehicle crashes.
